# Suppression of nitric oxide production from nasal fibroblasts by metabolized clarithromycin *in vitro*

**DOI:** 10.1186/1476-9255-7-56

**Published:** 2010-11-23

**Authors:** Ayako Furuya, Kazuhito Asano, Naruo Shoji, Kojiro Hirano, Taisuke Hamasaki, Harumi Suzaki

**Affiliations:** 1 Department of Otorhinolaryngology, School of Medicine, Showa University, Tokyo, Japan; 2 Division of Physiology, School of Nursing and Rehabilitation Sciences, Showa University, Yokohama, Japan

## Abstract

**Background:**

Low-dose and long-term administration of 14-membered macrolide antibiotics, so called macrolide therapy, has been reported to favorably modify the clinical conditions of chronic airway diseases. Since there is growing evidence that macrolide antibiotic-resistant bacteria's spreaders in the populations received macrolide therapy, it is strongly desired to develop macrolide antibiotics, which showed only anti-inflammatory action. The present study was designed to examine the influence of clarithromycin (CAM) and its metabolized materials, M-1, M-4 and M-5, on free radical generation from nasal polyp fibroblasts (NPFs) through the choice of nitric oxide (NO), which is one of important effector molecule in the development of airway inflammatory disease *in vitro*.

**Methods:**

NPFs (5 × 10^5 ^cells/ml) were stimulated with 1.0 μg/ml lipopolysaccharide (LPS) in the presence of agents for 24 hours. NO levels in culture supernatants were examined by the Griess method. We also examined the influence of agents on the phosphorylation of MAPKs, NF-κB activation, iNOS mRNA expression and iNOS production in NPFs cultured for 2, 4, 8, and 12 hours, respectively.

**Results:**

The addition of CAM (> 0.4 μg/ml) and M-4 (> 0.04 μg/ml) could suppress NO production from NPFs after LPS stimulation through the suppression of iNOS mRNA expression and NF-κB activation. CAM and M-4 also suppressed phosphorylation of MAPKs, ERK and p38 MAPK, but not JNK, which are increased LPS stimulation. On the other hand, M-1 and M-5 could not inhibit the NO generation, even when 0.1 μg/ml of the agent was added to cell cultures.

**Conclusion:**

The present results may suggest that M-4 will be a good candidate for the agent in the treatment of chronic airway inflammatory diseases, since M-4 did not have antimicribiological effects on gram positive and negative bacteria.

## Background

Macrolide antibiotics, such as roxithromycin and clarithromycin (CAM), are a well-established class of antibacterial agent, which are active against many species of Gram-positive and some Gram-negative bacteria. Besides their antibacterial activity, these compounds are reported to exert anti-inflammatory actions *in vitro *and *in vivo *[1-3]. It has been reported previously that macrolides suppress the inflammatory steps through the inhibition of inflammatory cell migration, modulation of oxidative burst and inflammatory cytokine production [4-6]. In addition, macrolides have beneficial effects in the treatment of chronic airway inflammatory diseases, such as diffuse panbronchiolitis (DPB), chronic sinusitis (CS) and cystic fibrosis [2]. In this regard, the anti-inflammatory action, but not the antimicrobial action of macrolides, is reported to be responsible for the clinical effectiveness of these agents against the inflammatory diseases [1,2,6-8]. On the other hand, since there is growing evidence that macrolide antibiotic-resistant bacteria's spreaders in the populations, who are orally administered macrolide antibiotics for long periods, it is strongly desired to develop macrolide antibiotics, which showed only anti-inflammatory action [9,10]. From that point of view, several types of derivatives of macrolide antibiotics were synthesized from erythromycin (EM) and their biological activities were examined *in vitro *and *in vivo*. Among these derivatives, EM201, obtained by mild acid treatment of EM, known as an internal metabolite of EM, has been reported to show a strong inhibitory effect on macrophage differentiation and to possess weak antimicrobial activity [11]. Furthermore, EM703, the 12-membered psuedoerythromycin A, was also reported to inhibit macrophage activation and to be free of any antibacterial activity, and was known to exert a prophylactic effect on lung injury in the rat model, similar to EM [12], suggesting that these derivatives from EM will be good candidates for drugs used in the treatment of airway inflammatory diseases.

Nitric oxide (NO), which was first identified as an endothelium-derived relaxing factor, is accepted as one of the important regulators of many cell and tissue functions. NO is also known to be produced by various types of cells and tissues (e.g. macrophages, epithelium and fibroblasts) in response to inflammatory stimulation [13]. Although physiological production of NO is generally believed to play an important role in host defense, overproduction of NO and its metabolites has been implicated in the pathogenesis of conditions such as bacterial sepsis, chronic inflammation [14] and pulmonary fibrosis [15].

After oral administration of CAM, the agent was metabolized into several types of metabolized materials, M-1, M-4 and M-5, among others [16]. In these materials, M-1 and M-5 show anti-microbial effects similar to that observed in CAM, whereas M-4 has no antibacterial effects [17]. Our previous work clearly shows the suppressive effects of M-4 on dendritic cell functions, such as inflammatory cytokine production and co-stimulatory molecule expression [18]. It is also observed that M-4 could inhibit the production of IL-8 from BEASE-2B cells, human airway epithelial cell line, in response to TNF-α stimulation *in vitro *[19]. However, the influence of M-4 on NO production is not still defined. In the present study, therefore, we examined whether M-4 could suppress NO production from nasal fibroblasts in response to inflammatory stimulation *in vitro*.

## Methods

### Agents

CAM and its metabolized materials, M-1, M-4 and M-5, are kindly donated by Taisho-Toyama Pharmaceutical Co. Ltd. (Osaka, Japan) as a preservative-free pure powder. They were firstly dissolved in 100% methanol at a concentration of 2.0 mg/ml, and then diluted with minimum essential medium (MEM; SIGMA Chemicals, St Louis, MO) supplemented with 3% heat-inactivated calf serum (MEM-FCS; Irvine, Santa Ana, CA) to give a concentration of 100.0 μg/ml. The solutions were then sterilized by passing through 0.2 μm filters and stored at 4°C as stock solutions. Lipopolysaccharide (LPS) extracted from *Escherichia coli *(SIGMA Chemicals) was dissolved in MEM-FCS at a concentration of 10.0 mg/ml. It was then sterilized by passing it through a 0.2 μm filter and diluted with MEM-FCS at appropriate concentrations for experiments.

### Cell source

Nasal polyp specimens were surgically obtained from chronic sinusitis patients who had not received any medical treatment, including systemic and topical steroid application. Specimens were cut into small pieces (approximately 1 mm) and washed several times in phosphate-buffered saline supplemented with 200 U/ml penicillin, 200 μg/ml streptomycin and 5.0 μg/ml amphotericin B, followed by MEM that contained 10% FCS. Diced specimens were then plated at a density of 10 pieces in 100 mm tissue culture dishes and covered with a cover slip adhered to the dish. The dishes were then placed at 37°C in a humidified atmosphere containing 5% CO_2_. When a monolayer of fibroblast-like cells was found to be confluent, the explanted tissues were removed. The cells were then trypsinized and replated at a concentration of 5 × 10^5 ^cells/ml. The medium (MEM containing 10% FCS) was changed every 3 days for 2-3 weeks until confluence was attained. Subsequently, the cells were split into two at confluence and passaged. The cells were characterized [20], and used as nasal polyp fibroblasts (NPFs). All donors (5 subjects) were male, aged between 25 and 62 years (mean 40.5 years) and had given their informed consent, according to the protocol approved by the Ethics Committee of Showa University.

### Cell culture

The cells, passaged 3-5 times, were washed several times with MEM-FCS, introduced into each well of 24-well culture plates in triplicate at a concentration of 5 × 10^5 ^cells/ml in a volume of 1.0 ml and allowed to adhere for 2 hours. The plates were then washed twice with MEM-FCS to remove dead and unattached cells. The residual cells were stimulated with LPS in the presence of various concentrations of agents in a total volume of 2.0 ml. To prepare culture supernatants, cells were cultured for 24 hours [21], and the culture medium was removed and stored at -40°C until used. Cells for examination of phosphorylation of mitogen-activated protein kinases (MAPKs), transcription factor activation, inducible NO synthase (iNOS) mRNA expression and iNOS protein were cultured in a similar manner for 2, 4, 8 and 12 hours, respectively. The cells were then stored at -80°C and used within 24 hours. In all experiments, treatment of cells with the agents was started 2 hours before LPS stimulation.

### Assay for cell proliferation

Cell proliferation induced by LPS stimulation was examined by a commercially available Cell Proliferation enzyme-linked immunosorbent assay (ELISA) test kit (GE Healthcare Ltd., Buckinghamshire, UK) that contained sufficient reagents according to the manufacturer's recommended procedures. Briefly, cells (1 × 10^5 ^cells/well) stimulated with LPS for 48 hours in the presence of various concentrations of CAM and M-4 in 96-well flat-bottomed culture plates in triplicate were labeled with 10 μM 5-brom-2'-deoxyuridine (BrdU) for 2 hours. After removing BrdU solution, cells were blocked with blocking buffer for 30 min and then treated with peroxidase-labeled anti-BrdU monoclonal antibody for 90 min. After washing three times with washing buffer, 3,3'5,5'-tetramethylbenzidine (TMB) was added into each well and incubated for 30 min. After addition of 1 M sulphuric acid, the optical density (OD) at 450 nm was measured with an ELISA plate reader. The results were expressed as the mean OD ± SE of five different subjects.

### Assay for NO (NO_2_^-^/NO_3_^-^)

The NO concentration in culture supernatants was measured using Griess Reagents Kits for NO_2_^-^/NO_3_^- ^assay (Dojindo, Co. Ltd., Kumamoto, Japan). The assay was done in duplicate, and the results were expressed as the mean μM ± SE of five different subjects.

### Assay for inducible NO synthases (iNOS)

The iNOS levels in cytosol were assayed by commercially available human iNOS ELISA kits (R & D Systems, Inc., Minneapolis, MN) that contained sufficient reagents, according to the manufacturer's recommendations. Samples used for examining iNOS levels were prepared from 5 × 10^5 ^cells cultured for 12 hours. The results were expressed as the mean U/ml ± SE of five different subjects. The minimum detectable level of this ELISA kit was 0.15 U/ml.

### Assay for iNOS mRNA expression

iNOS mRNA was examined using commercially available ELISA test kits for human iNOS mRNA that contained sufficient reagents, according to the manufacturer's recommendations. Poly A^+ ^mRNA was separated from cells cultured for 8 hours using oligo(dT)-coated magnetic microbeads (Milteny Biotec, Bergisch Gladbach, Germany), and used as target mRNA at a concentration of 2.0 μg for examining iNOS mRNA expression. Poly A^+ ^mRNA in a volume of 150 μl were added into each well of a 96-well microplate that contained 50 μl of specific probe in duplicate and incubated for 60 min at 65°C. The materials (150 μl) were then transferred into each well of a 96-well microplate, which was coated with streptavidin and incubated for 60 min at 25°C. Polyclonal antibody against digoxigen conjugated to alkaline phosphatase was added to wells and incubated at 25°C. After 60 min, 50 μl of NADPH solution was added and incubated for 60 min. After addition of enzymes, OD at 490 nm was measured, and the results were expressed as the mean OD ± SE of five different subjects.

### Assay for transcription factor activation

Nuclear factor-κB **(**NF-κB) activity was analyzed using a commercially available ELISA test kits (Active Motif, Co., Ltd, Carlsbad, CA), which contained sufficient reagents and monoclonal antibodies against p50 subunit, according to the manufacturer's recommendations. Briefly, nuclear extract (5.0 μg protein) from 4-hour cultured cells was introduced into each well of a 96-well microplate precoated with oligonucleotide containing the NF-κB consensus site (5'-GGGACTTTCC-3') in a volume of 20 μl, and incubated for 1 hour at 25°C. After washing three times, 100 μl monoclonal antibody against p50 was added to the appropriate wells, and incubated for a further 1 hour at 25°C. Anti-IgG HRP-conjugate in a volume of 100 μl was then added and incubated for 1 hour at 25°C. OD at 450 nm was measured after the addition of tetramethylbenzyne solution. Using the manufacturer's data sheets, the amount of NF-κB bound to DNA can be measured by this ELISA system. ELISA was done in duplicate, and the results were expressed as the mean OD ± SE of five different subjects.

### Assay for phosphorylation of MAPKs

The phosphorylation of p38 MAPK was measured by a commercially available ELISA test kit (Active Motif, Co. Ltd) according to the manufacturer's recommendations. Briefly, cells cultured for 2 hours in 96-well culture plates were fixed with 4% formaldehyde for 20 min at 25°C. After washing three times, 100 μl antibody blocking buffer was added into each well, and incubated for 1 hour at 25°C. After removing blocking buffer, 40 μl primary antibody (phosphorylated-p38 MAPK antibody) was added, and incubated for a further 12 hours at 4°C. Secondary antibody (anti-IgG HRP-conjugate) was added in a volume of 100 μl, and incubated for 1 hour at 25°C. OD at 450 nm was measured after the addition of tetramethylbenzyne solution. The phosphorylation of both extracellular signal related kinase (ERK)1/2 and Jun N-terminal kinase (JNK) were also measured with ELISA test kits (Active Motif, Co. Ltd.) in a similar manner. In all phosphorylation assay, ELISA was done in duplicate, and the results were expressed as the mean OD ± SE of five different subjects.

### Statistical evaluation

A one-way ANOVA test was employed for statistical analysis, with significant difference determined as P < 0.05.

## Results

### Suppression of NO production from NPFs by CAM and its metabolized materials

**Figure 1:**
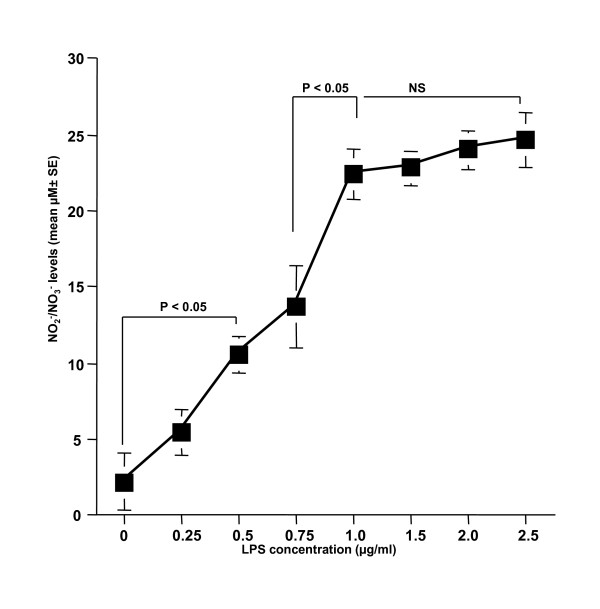
**Influence of LPS stimulation on NO production from NPFs**. NPFs at a concentration of 5 × 10^5 ^cells were stimulated with various concentrations of LPS. After 24 hours, culture supernatants were obtained and assayed for NO (NO_2_^-^/NO_3_^-^) levels by the Griess method. Data are the mean ± SE of five different subjects. LPS, lipopolysaccharide; NO, nitric oxide; NPFs, nasal polyp fibroblasts. NS, not significant (P > 0.05).

**Figure 2:**
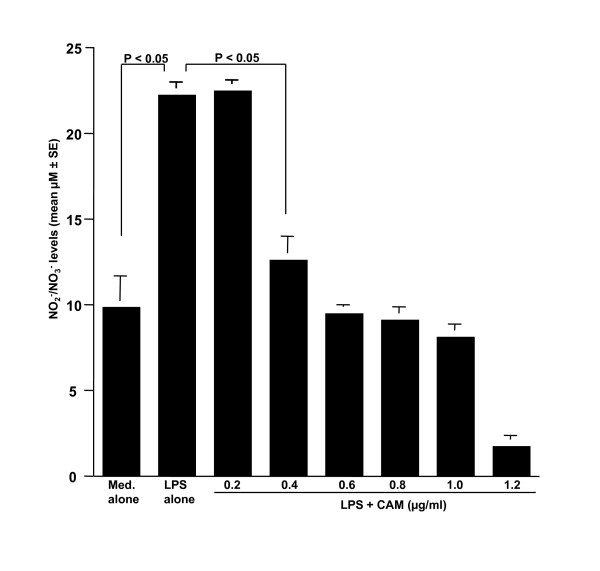
**Influence of CAM on NO production from NPFs in response to LPS stimulation**. NPFs at a concentration of 5 × 10^5 ^cell/ml were stimulated with 1.0 μg/ml LPS in the presence of various concentrations of CAM. After 24 hours, culture supernatants were obtained and NO (NO_2_^-^/NO_3_^-^) levels were assayed by the Griess method. Data are the mean ± SE of five different subjects. CAM, clarithromycin; NO, nitric oxide; NPFs, nasal polyp fibroblasts; LPS, lipopolysaccharide.

**Figure 3:**
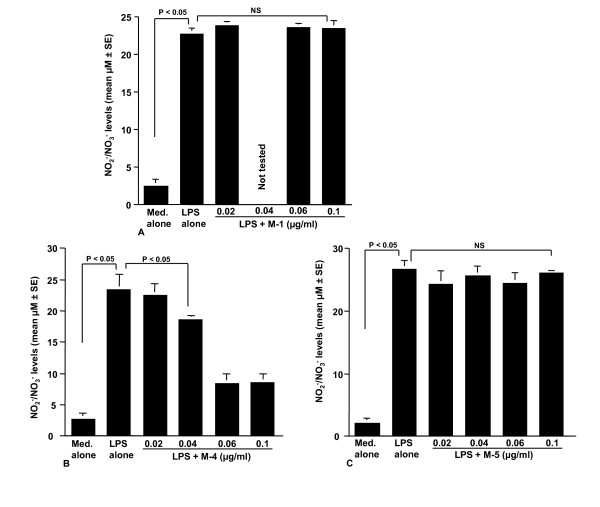
**Influence of metabolized clarithromycin, M-1 (A), M-4 (B) and M-5 (C) on NO production from NPFs in response to LPS stimulation**. NPFs at a concentration of 5 × 10^5 ^cell/ml were stimulated with 1.0 μg/ml LPS in the presence of various concentrations of the agents. After 24 hours, culture supernatants were obtained and NO (NO_2_^-^/NO_3_^-^) levels were assayed by the Griess method. Data are the mean ± SE of five different subjects. NO, nitric oxide; NPFs, nasal polyp fibroblasts; LPS, lipopolysaccharide; NS, not significant (P > 0.05).

The first set of experiments was undertaken to examine the influence of LPS stimulation on NO production from NPFs. NPFs were stimulated with various concentrations of LPS in triplicate and the culture supernatants were collected 24 hours later for measurement of NO concentration. As shown in Figure 1, LPS stimulation caused a dose-dependent increase in NO production from NPFs, which was first detected at 0.5 μg/ml and peaked at more than 1.0 μg/ml. We then examined the influence of CAM on NO production from NPFs in response to LPS stimulation. NPFs were stimulated with 1.0 μg/ml LPS in the presence of various concentrations of CAM for 24 hours. The addition of CAM into cell cultures caused suppression of NO production (Figure 2). The minimum concentration of CAM, which caused significant suppression of NO production was 0.4 μg/ml (Figure 2). The third set of experiments was designed to examine the influence of metabolized CAM, M-1, M-4 and M-5, on NO production from NPFs induced by LPS stimulation. As shown in Figure 3A, M-1 could not inhibit NO production from NPFs, even when 0.1 μg/ml of the agent was added to cell cultures. On the other hand, the addition of M-4 at more than 0.04 μg/ml exerted the suppressive effect on NO production from NPFs (Figure 3B). The data in Figure 3C also showed the negative suppressive effect of M-5 at 0.1 μg/ml on NO production from NPFs: NO levels in culture supernatants from cells treated with 0.1 μg/ml M-5 were similar to that from control supernatants (P > 0.05).

### Influence of CAM and M-4 on cell proliferation induced by LPS stimulation

**Figure 4:**
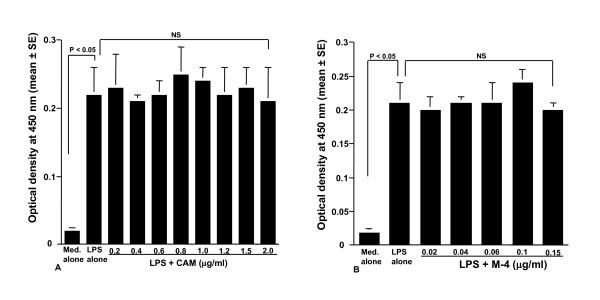
**Influence of CAM (A) and M-4 (B) on cell proliferation induced by LPS stimulation**. NPFs at a concentration of 1 × 10^5 ^cells/ml were stimulated 1.0 μg/ml LPS in the presence of various concentrations of CAM and M-4. After 48 hours, cell proliferation was examined by ELISA. Data are the mean OD at 450 nm ± SE of five different subjects. LPS, lipopolysaccharide; NPFs, nasal polyp fibroblasts; CAM, clarithromycin; CAM, clarithromycin; ELISA, enzyme-linked immunosorbent assay; OD, optical density; NS, not significant (P > 0.05).

The fourth set of experiments was carried out to examine the influence of CAM and M-4 on cell proliferation induced by LPS stimulation. NPFs were stimulated with 1.0 μg/ml LPS in the presence of various concentrations of CAM and M-4 for 48 hours. Cell proliferation was examined by ELISA. As shown in Figure 4A, addition of CAM into cell cultures scarcely affected cell proliferation and OD at 450 nm in experimental groups was similar (not significant; P > 0.05) to that observed in cells stimulated with LPS alone. The data in Figure 4B also showed that M-4 did not exert harmful effects on cell proliferation induced by LPS stimulation: OD at 450 nm in cells treated with M-4 at 0.15 μg/ml was nearly identical (not significant; P > 0.05) to that observed in LPS alone.

### Influence of CAM and M-4 on iNOS levels in NPFs after LPS stimulation

**Figure 5:**
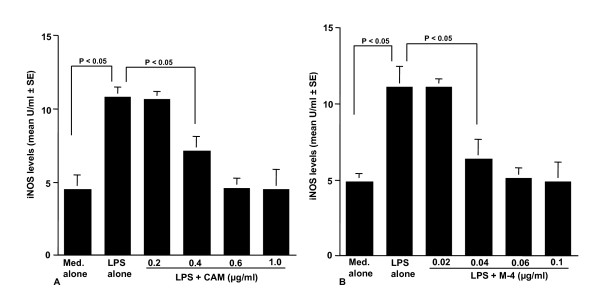
**Influence of CAM (A) and M-4 (B) on iNOS production in NPFs after LPS stimulation**. NPFs at a concentration of 5 × 10^5 ^cells/ml were stimulated 1.0 μg/ml LPS in the presence of various concentrations of CAM and M-4. After 12 hours, NPFs were collected and iNOS levels were assayed by ELISA. Data are the mean ± SE of five different subjects. iNOS, inducible nitric synthase; NPFs, nasal polyp fibroblasts; LPS, lipopolysaccharide; CAM, clarithromycin; ELISA, enzyme-linked immunosorbent assay.

The fifth set of experiments was done to examine the influence of CAM and M-4 on iNOS production from NPFs after LPS stimulation. NPFs were stimulated with 1.0 μg/ml LPS in the presence or absence of the agents for 12 hours. iNOS levels in cytosol were examined by ELISA. As shown in Figure 5A, the addition of CAM at more that 0.4 μg/ml into cell cultures caused significant suppression of iNOS levels in NPFs, which was increased by LPS stimulation. The data in Figure 5B also showed that M-4 at more than 0.04 μg/ml, but not 0.02 μg/ml, could exert suppressive effects on the increase in iNOS levels in NPFs after LPS stimulation.

### Influence of CAM and M-4 on iNOS mRNA expression

**Figure 6:**
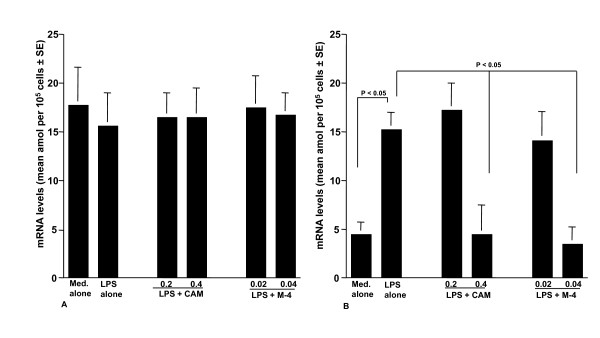
**Influence of clarithromycin (A) and M-4 (B) on iNOS mRNA expression in NPFs after LPS stimulation**. NPFs at a concentration of 5 × 10^5 ^cells/ml were stimulated 1.0 μg/ml LPS in the presence of various concentrations of CAM and M-4. After 8 hours, Poly A^+ ^was obtained from NPFs and iNOS mRNA levels were assayed by ELISA. Data are the mean ± SE of five different subjects. iNOS, inducible nitric synthase; NPFs, nasal polyp fibroblasts; LPS, lipopolysaccharide; CAM, clarithromycin; ELISA, enzyme-linked immunosorbent assay.

The sixth set of experiments was undertaken to examine the influence of CAM and M-4 on iNOS mRNA expression in NPFs after LPS stimulation. NPFs were stimulated with LPS in the presence of CAM and M-4 for 6 hours. iNOS mRNA expression was examined by ELISA. The addition of CAM and M-4 into cell cultures scarcely affected GAPDH mRNA expression in NPFs cultured for 8 hours (Figure 6A), whereas iNOS mRNA expression was significantly suppressed by CAM and M-4, when these agents were added to cell cultures at 0.4 μg/ml and 0.04 μg/ml, respectively (Figure 6B).

### Assay for CAM and M-4 on NF-κB activation and phosphorylation of MAPKs

**Figure 7:**
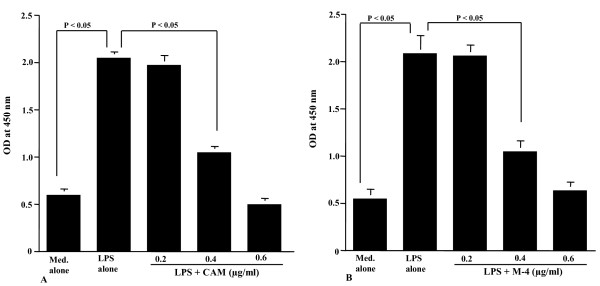
**Influence of clarithromycin (A) and M-4 (B) on NF-κB activation in NPFs after LPS stimulation**. NPFs at a concentration of 5 × 10^5 ^cells/ml were stimulated 1.0 μg/ml LPS in the presence of various concentrations of clarithromycin (CAM) and M-4. After 4 hours, nuclear extracts were obtained from NPFs and NF-κB p50 activity was assayed by ELISA. Data are the mean ± SE of five different subjects. NF-κB, nuclear factor-κB; NPFs, nasal polyp fibroblasts; LPS, lipopolysaccharide; CAM, clarithromycin; ELISA, enzyme-linked immunosorbent assay.

**Figure 8:**
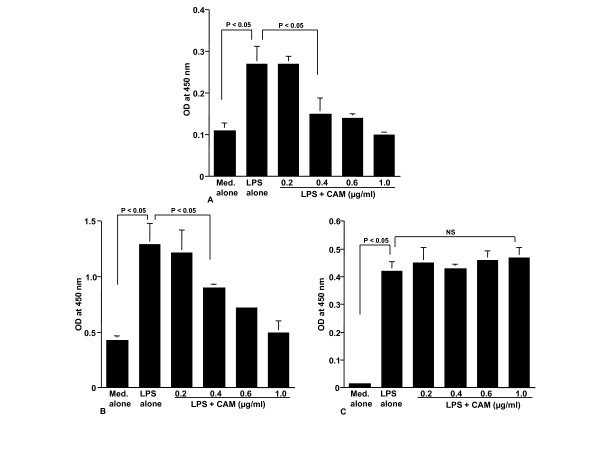
**Influence of clarithromycin on MAPKs activation in NPFs after LPS stimulation**. NPFs at a concentration of 5 × 10^5 ^cells/ml were stimulated 1.0 μg/ml LPS in the presence of various concentrations of CAM. After 4 hours, MAPKs activation was assayed by ELISA. Data are the mean ± SE of five different subjects. A, p38 MAPK; B, ERK1/2; C, JNK. MAPKs, mitogen-activated kinases; NPFs, nasal polyp fibroblasts; LPS, lipopolysaccharide; CAM, clarithromycin; NS, not significant (P > 0.05).

**Figure 9:**
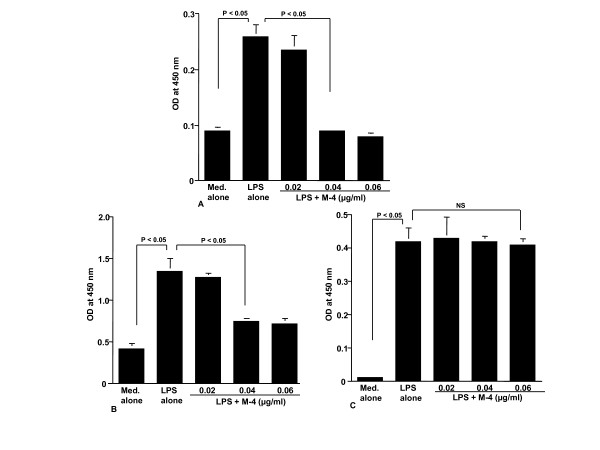
**Influence of metabolized clarithromycin, M-4, on MAPKs activation in NPFs after LPS stimulation**. NPFs at a concentration of 5 × 10^5 ^cells/ml were stimulated 1.0 μg/ml LPS in the presence of various concentrations of M-4. After 4 hours, MAPKs activation was assayed by ELISA. Data are the mean ± SE of five different subjects. A, p38 MAPK; B, ERK1/2; C, JNK. MAPKs, mitogen-activated kinases; NPFs, nasal polyp fibroblasts; LPS, lipopolysaccharide; ELISA, enzyme-linked immunosorbent assay; NS, not significant (P > 0.05).

The final set of experiments was undertaken to examine the influence of CAM and M-4 on transcription factor activation and signal transduction pathways in NPFs after LPS stimulation. To do this, NPFs were stimulated with LPS in the presence of either CAM or M-4 for 2 hours. NF-κB activation and phosphorylation of MAPKs were examined by ELISA. NF-κB activation in NPFs, which was enhanced by LPS stimulation decreased by the treatment of cells with CAM (Figure 7A) and M-4 (Figure 7B). The minimum concentrations of these agents, which caused significant suppression, were 0.4 μg/ml for CAM (Figure 7A) and 0.04 μg/ml for M-4 (Figure 7B). We then examined the influence of CAM and M-4 on phosphorylation of MAPKs, p38 MAPK, ERK1/2 and JNK in NPFs cultured for 2 hours with LPS. Treatment of NPFs with CAM at more than 0.4 μg/ml could inhibit the increase in phosphorylation of both p38 MAPK (Figure 8A) and ERK1/2 (Figure 8B) induced by LPS stimulation. However, CAM could not inhibit JNK phosphorylation by LPS stimulation, even when 1.0 μg/ml CAM was used for the NPFs treatment: OD at 450 nm in cells treated with 1.0 μg/ml CAM was nearly identical (P > 0.05) to that observed in cells treated with LPS alone (Figure 8C). We finally examined the influence of M-4 on MAPKs phosphorylation in NPFs after LPS stimulation. Treatment of cells with M-4 also caused inhibition of phosphorylation of both p38 MAPK (Figure 9A) and ERK1/2 (Figure 9B) in NPFs stimulated with LPS and the minimum concentration of the agent, which caused significant suppression was 0.04 μg/ml (Figure 9A and 9B)). On the other hand, M-4 at 0.06 μg/ml could not inhibit JNK phosphorylation in NPFs induced by LPS stimulation (Figure 9C).

## Discussion

Low-dose and long-term administration of macrolide antibiotics, so called macrolide therapy, is effective in the treatment of upper and lower airway chronic inflammatory diseases, such as DPB and CS, if the patient is administered 14- and 15-membered macrolides (e.g. CAM and azithromycin), but not 16-membered macrolide, including josamycin [2]. There is considerable evidence to suggest that the anti-inflammatory action of macrolides, such as the inhibition of inflammatory cytokine production and polymorphonuclear leukocyte activation, may account for the clinical effectiveness of macrolides in inflammatory airway diseases [1,3-6,8]. Recently, free radicals have attracted attention as important final effector molecules in inflammatory diseases, including DPB and CS [13,14,21], whereas the influence of macrolides on free radical generation is not well defined.

It is now accepted that polymorphonuclear leukocytes play essential roles in the development of inflammatory responses via the production of several types of chemical mediators and inflammatory cytokines [5]. Reactive oxygen species such as O_2_^- ^and H_2_O_2 _are also produced from polymorphonuclear leukocytes and are responsible for the modification of inflammatory responses [5]. In addition to O_2_^- ^and H_2_O_2_, another reactive oxygen species, NO, is also well known to be involved in the pathogenesis of inflammatory processes [15,22,23]. NO generated from a number of cells (e.g. immune cells and fibroblasts) after inflammatory stimulation is rapidly oxidized to it's more stable metabolites: nitrite and nitrate [13,23]. Nitrite and nitrate are then reacted with superoxide to produce the very reactive and toxic peroxynitrite, which can initiate lipid peroxidation on the outer cell membrane and tissue injury [13,23]. NO also can easily diffuse across the cell membrane and reacts with intracellular superoxide to form peroxynitrite, which causes nuclear membrane and DNA damage in inflammatory tissues [24]. In a study performed in rabbits, elevated NO metabolite, nitrite and nitrate, levels were founded in lavage fluid from chronic sinusitis and returned to normal levels during recovery [25]. In human cases, NO metabolite levels in sinus lavage fluid were also reported to be significantly increased in chronic rhinosinusitis compared with normal sinus [26]. Further more, the sputum obtained from patients with cystic fibrosis is reported to contain much higher levels of nitrite/nitrate compared with that from normal subjects, and these levels correlate with disease exacerbation [27]. The present results clearly showed that CAM could exert the suppressive effect on the ability of NPFs to produce NO in response to LPS stimulation when the cells were treated with the agent at more than 0.4 μg/ml, which is quite low levels compared with therapeutic blood levels (1.03 ± 0.16 μg/ml) [16]. It is also observed that this suppressive effect of CAM on NO production is not owing to its lethal effect on NPFs: LPS-induced proliferation of NPFs treated with CAM at 2.0 μg/ml is quite similar to that observed in non-treated control. Taken together, the present results strongly suggest that the suppressive effect of CAM on NO production may underlie the therapeutic mode of action of the agent on inflammatory airway diseases, including CS. This speculation may be supported by the observation that oral administration of macrolide antibiotics such as roxithromycin and azithromycin into mice once a day for 4 weeks significantly suppress NO generation induced by LPS injection [28]. Pretreatment of mice with telithromycin, one of ketolide antibiotics derived from 14-membered macrolide antibiotics, as well as roxithromycin has been reported to attenuate LPS-induced acute systemic inflammation through the suppression of iNOS mRNA expression and NO production [29]. This observation also support our speculation that suppressive effect of CAM on NO production from fibroblasts may be one of the mechanisms leading to the favorable modification of airway inflammation as a result of macrolide therapy. It is reported that after oral administration of CAM into human, the agent is metabolized into several types of metabolized materials, including M-1, M-4 and M-5, among others [16,17]. Among these materials, M-5 shows strong antimicrobial effects similar to that of non-metabolized CAM [17]. Other materials show extremely low antibacterial activity and M-4 has no antibacterial effects [17]. It is strongly desired to develop macrolide antibiotics, which show only immuno-modulatory effects [9,10]. These reports prompted us to explore the influence of metabolized CAM on NO production from fibroblasts *in vitro*. The present data clearly showed that M-1 and M-5 did not show the inhibitory action of NO production, even when 0.1 μg/ml, twice that of therapeutic blood levels [16] were added to cell cultures. On the other hand, the addition of M-4 at 0.04 μg/ml, which is a tenth of CAM, caused significant suppression of NO production from fibroblasts, suggesting that M-4 may be responsible for improving clinical conditions of inflammatory airway diseases, including CS, through the suppression of NO production. The present results also suggest that M-4 will be a good candidate as the agent used for the treatment of airway inflammatory diseases, since M-4 does not show any antimicrobial activity [17].

NO is primarily derived from a cationic amino acid, L-arginine, and oxygen by a family of NOS. To date, three NOS isoforms, neural NOS (nNOS), endothelial NOS (eNOS) and iNOS, have been identified [11]. Among these NOS, iNOS that is generally not present in quiescent cells is often induced by inflammatory stimuli and mediates high levels of NO generation for long periods, resulting in tissue injury and mutations in cells [13,23,24]. Recent reports have clearly showed that macrolide antibiotics such as telithromycin and roxithromycin inhibit NO generation through the suppression of iNOS mRNA expression *in vitro *and *in vivo *[28-30]. These reports open the questions of whether CAM and M-4 on NO production is due to their inhibitory action of iNOS generation by iNOS mRNA expression or their suppression of iNOS activity to produce NO. We then examined the influence of CAM and M-4 on iNOS mRNA expression in fibroblasts. Our data clearly showed the suppressive activity of CAM and M-4 on iNOS generation through the inhibition of iNOS mRNA expression in NPFs, which was enhanced by LPS stimulation. It is reported that the induction of excess iNOS in endothelial cells causes cell injury and inhibits cellular respiration, which leads to cell dysfunction and cell death [21]. It is also observed that iNOS could produce significant amounts of superoxide, which is responsible for the formation of the most toxic molecules, hydrogen radicals [21]. Furthermore, down-regulation of iNOS expression suppresses the production of inflammatory cytokines as well as matrix metalloproteinases, which are essential molecules for the development of CS [3], suggesting that CAM administered orally and M-4 synthetized from CAM cause a decrease in iNOS expression in cytosol after inflammatory stimulation, inhibiting superoxide generation and resulting in prevention of tissue injury in patients with chronic airway diseases, including CS.

The cellular response to LPS is transmitted from the cell membrane to the cytoplasm through the Toll-like receptor 4 in concert with CD14 and lipid binding protein. In turn, many signal transduction pathways and transcription factors, especially NF-κB that are essential for iNOS mRNA expression after inflammatory stimulation are activated [31,32]. LPS stimulates the phosphorylation and activation of three types of MAPKs, ERK1/2, p38 MAPK and JNK, which are essential for iNOS mRNA expression after inflammatory stimulation [33-35]. Therefore, we performed final experiments to explore the possible mechanisms by which CAM and M-4 could suppress iNOS mRNA expression in NPFs in response to LPS stimulation. The present data clearly showed that CAM and M-4 exert suppressive effects on NF-κB activation and phosphorylation of MAPKs, ERK1/2 and p38 MAPK in fibroblasts after LPS stimulation. On the other hand, treatment of fibroblasts with CAM and M-4 could not inhibit JNK phosphorylation, indicating that this type of MAPK is not required for iNOS mRNA induction in NPFs after LPS stimulation. In addition to these signaling pathways, the cyclic AMP (cAMP)/protein kinase A (PKA) pathway has been reported to mediate iNOS expression after LPS stimulation: activation of the cAMP/PKA pathway inhibited LPS-induced iNOS expression in murine macrophages and hepatocytes [36,37]. The protein kinase C (PKC) pathway is also reported to be involved in LPS-induced iNOS expression, and activation of PKC isoforms, especially PKC-α, diminished iNOS expression in macrophages after LPS stimulation [38]. These reports may suggest that CAM and M-4 affect cAMP/PKA and PKC pathways to suppress expression of iNOS mRNA in NPFs after LPS stimulation. Further experiments are required to clarify this point. Stimulation of cells with LPS causes an increase in intracellular Ca^2+ ^concentration by opening the voltage-gated calcium channels [39]. It is reported that Ca^2+ ^is an essential molecule for phosphorylation of MAPKs, which is responsible for activation of NF-κB [40], after stimulation of cells with inflammatory mediators such as LPS and TNF-α [39,40]. Erythromycin is also reported to inhibit an increase in Ca^2+ ^concentration through the suppression of Ca^2+ ^influx from the extracellular space [41]. These reports may suggest that treatment of NPFs with CAM and M-4 caused inhibition of the increase in intracellular Ca^2+ ^concentration induced by LPS stimulation, and result in suppression of phosphorylation of MAPKs such as p38 MAPK and ERK1/2 and NF-κB activation.

## Conclusions

Our results strongly suggest that inhibitory action of CAM and M-4 on NO production constitutes at least part of the therapeutic mode of action of the agent on chronic airway inflammatory diseases. It is also suggested that M-4 will be a good candidate for the treatment of chronic airway diseases, since this agent was free of any antibacterial activity.

## Competing interests

The authors declare that they have no competing interests.

## Authors' contributions

KA contributed to the concept and design of the study, and to the manuscript writing. AF, NS, KH and TH performed surgical operation and cell culture. KA examined NO levels, iNOS expression, and assayed phosphorylation of MAPKs, NF-κB activity. HS contributed the data analysis and to the manuscript writing. All authors read and approved the final manuscript.
